# An Unfortunate Polyneuropathy, Organomegaly, Endocrinopathy, Monoclonal Gammopathy, and Skin Change (POEMS)

**DOI:** 10.7759/cureus.1086

**Published:** 2017-03-08

**Authors:** Faraz Afridi, Jorge Otoya, Samantha F Bunting, Gerard Chaaya

**Affiliations:** 1 Internal Medicine, University of Central Florida College of Medicine; 2 Hematology and Medical Oncology, Osceola Regional Medical Center; 3 University of Central Florida College of Medicine

**Keywords:** poems syndrome, plasma cell leukemia

## Abstract

POEMS syndrome is an acronym for polyneuropathy, organomegaly, endocrinopathy, monoclonal gammopathy and skin changes, which is a rare paraneoplastic disease of monoclonal plasma cells. A mandatory criterion to diagnose POEMS syndrome is the presence of a monoclonal plasma cell dyscrasia in which plasma cell leukemia is the most aggressive form. Early identification of the features of the POEMS syndrome is critical for patients to identify an underlying plasma cell dyscrasias and to reduce the morbidity and mortality of the disease by providing early therapy. We present a case of a 64-year-old male who presented with non-specific symptoms and was found to have primary plasma cell leukemia, which was part of his unfortunate POEMS syndrome.

## Introduction

Polyneuropathy, organomegaly, endocrinopathy, monoclonal gammopathy, and skin changes (POEMS) syndrome, also known as Crow-Fukase syndrome is a rare paraneoplastic disease of monoclonal plasma cells which was first reported in 1956 [1]. The acronym of “POEMS” was derived in 1980 by Bardwick and colleagues on the basis of the five unique characteristic features [2]. The POEMS syndrome can present with plasma cell dyscrasias and therefore early identification of this syndrome may aid in the clinical course of plasma cell leukemias and other plasma cell proliferative disorders. Informed consent statement was obtained for this study.

## Case presentation

A 64-year-old Caucasian male with a past medical history of hypertension, diabetes mellitus, peripheral neuropathy and recently diagnosed primary hypogonadism presented due to a two-week history of gradual onset of shortness of breath at rest, fatigue and weight loss of 30 lbs in the last one month. He also reported worsening numbness of the lower extremities up to the abdomen despite excellent diabetic control. He denied chest pain, palpitations, cough, orthopnea, paroxysmal nocturnal dyspnea, lightheadedness, fevers, chills or change in stool or urinary symptoms. Social history was significant for only a smoking history of 14 pack years. The physical exam including vital signs was normal, except for splenomegaly and sensory neuropathy of the lower extremities up to the lower abdomen. 

**Figure 1:**
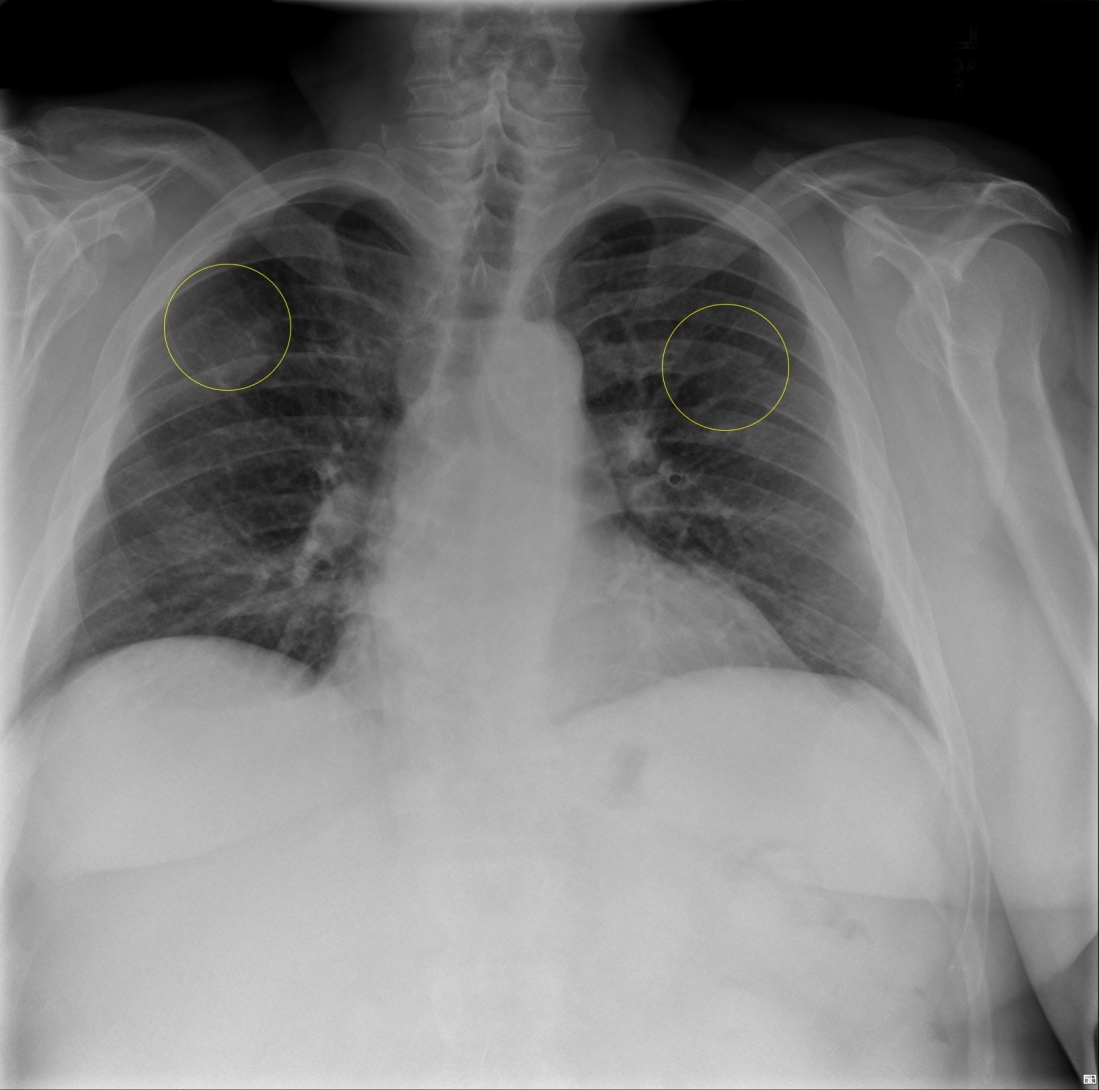
Chest x-ray showing multiple subacute rib fractures (yellow circles)

**Figure 2:**
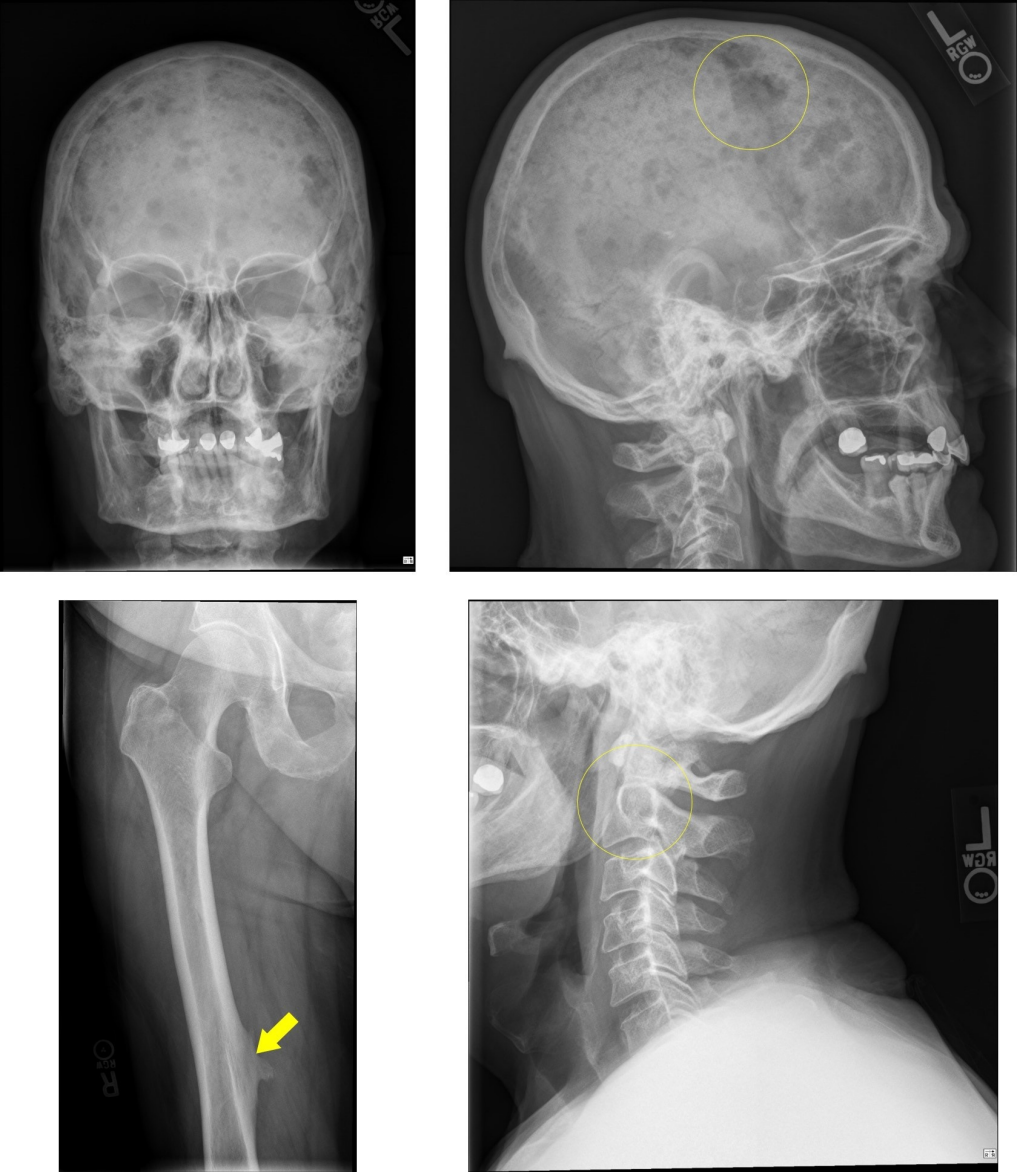
Multiple lytic bone lesions of the skull and cervical spine (largest in yellow circles) and a pathological lytic bone lesion of the right femur (yellow arrow)

The complete blood count showed new-onset normocytic anemia (hemoglobin of 9.0 g/dl, mean corpuscular volume of 86.7 fL), normal white blood cell count but with plasmacytosis (plasma cell count of three x 10^9^/L) on the differential cell count and thrombocytopenia (platelets of 129 x 10^_9_^/L). The complete metabolic profile showed an increased corrected calcium, aspartate amino transferase (AST), amino alanine transferase (ALT), alkaline phosphatase and total bilirubin (predominantly direct). Lactate dehydrogenase (LDH) level was normal but β2-macroglobulin and plasma vascular endothelial growth factor (VEGF) was elevated. A chest x-ray showed subacute fractures of multiple ribs (Figure 1). The skeletal survey revealed diffuse lytic bone lesions throughout the osseous structures of the skull and cervical spine and pathological fractures of the posterior ribs and femoral bones (Figure 2). 

**Figure 3:**
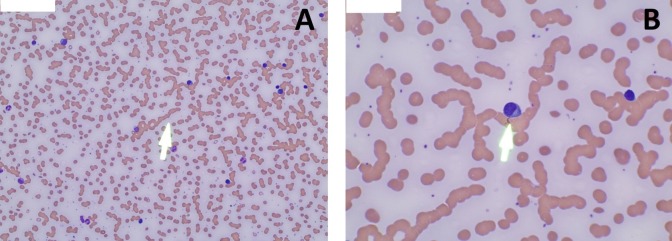
A: Peripheral blood smear showing rouleaux formation (white arrow); B: Peripheral blood smear showing plasma cells (white arrow)

**Figure 4:**
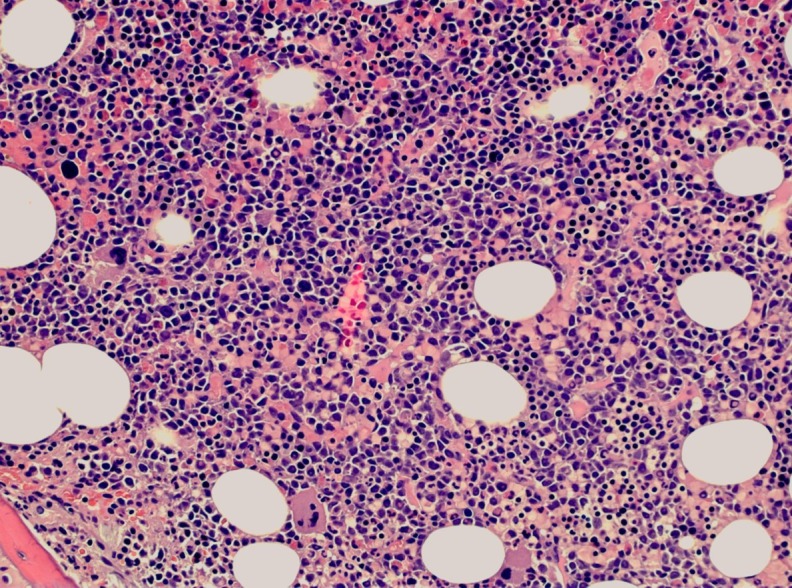
Bone marrow core biopsy showing a plasma cell infiltration of 90%

A peripheral blood smear showed rouleaux formation (Figure 3A) and numerous plasma cells (Figure 3B). Flow cytometry of the peripheral blood showed a 34% population of monoclonal plasma cells expressing CD36, CD138, and CD56 with a cytoplasmic kappa light chain restriction. The serum protein electrophoresis with immunofixation showed an IgA kappa monoclonal peak. The bone marrow biopsy showed a plasma cell infiltration of 90% (Figure 4). 

The cytogenetic studies of the bone biopsy were significant for monosomy 13. This workup was consistent with the diagnosis of primary plasma cell leukemia. 

The patient was started on intravenous fluids, prophylactic antibiotics and induction chemotherapy with a Velcade, Dexamethasone, Thalidomide (VDT-PACE regimen) replaced with lenalidomide because of peripheral neuropathy, cisplatin, adriamycin, cyclophosphamide, and etoposide. His course was complicated with liver failure from plasma cell infiltration and subsequent neutropenic fever, septic shock with multi-organ failure. Unfortunately, the patient expired three weeks after diagnosis.

## Discussion

Plasma cell leukemia (PCL) is the most aggressive plasma cell proliferative disorder. Primary PCL occurs de novo in patients with no evidence of previous multiple myeloma, whereas secondary PCL is an evolution of an underlying multiple myeloma. The incidence of PCL is between two percent to four percent of patients with multiple myeloma [3]. It is defined by the presence of more than 20% of plasma cells in the peripheral blood and an absolute plasma cell count greater than two x 10^9^/L. Prognosis is poor due to the high proliferative index, high tumor burden and rapid clinical course of the disease. The estimated median survival is estimated to be only four months [4], therefore early diagnosis and treatment may aid a better prognosis.

**Figure 5:**
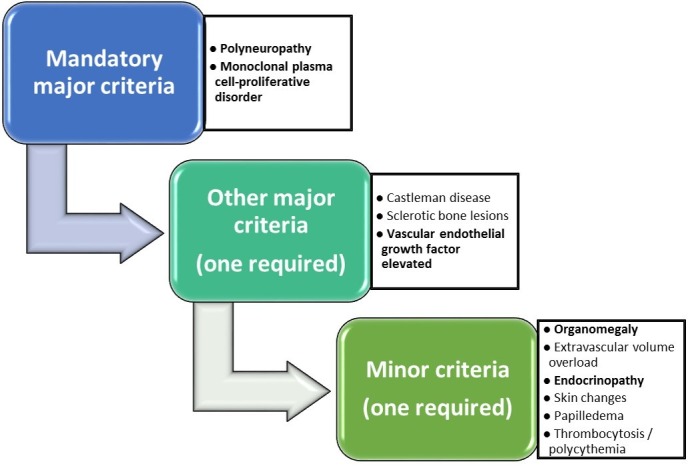
Diagnostic criteria for POEMS syndrome (those in bold were demonstrated by the patient)

POEMS syndrome is a rare paraneoplastic syndrome due to an underlying plasma cell disorder with an estimated prevalence of 0.3 per 100,000 people [5]. Other names that have been used to describe this rare entity include osteosclerotic myeloma, Takatsuki syndrome, and Crow-Fukase syndrome. The acronym of “POEMS” was derived in 1980 by Bardwick and colleagues on the basis of the five unique characteristic features: Polyneuropathy, Organomegaly, Endocrinopathy, Monoclonal gammopathy, and Skin changes [2]. Importantly, not all of the characteristic features in the acronym are required to make the diagnosis and there are other characteristic features of POEMS that are important. These include papilledema, volume overload, sclerotic bone lesions, thrombocytosis and erythrocytosis, increased vascular endothelial growth factor (VEGF) levels and a predisposition towards thrombosis. The pathogenesis of this syndrome is largely unknown but VEGF has been suggested to be a role in the pathogenesis as it is seen to be often elevated in patients with POEMS syndrome. VEGF is a cytokine which is expressed by osteoblasts, macrophages, plasma cells and platelets and functions to target endothelial cells, increase vascular permeability and angiogenesis. Although levels of VEGF has been correlated with disease activity of POEMS syndrome, the use of anti-VEGF therapy has been conflicting [6]. Clinical features include the dominant clinical sign of neuropathy which is peripheral, ascending, symmetrical and can affect both sensation and motor function and can sometimes present as hyperesthesia in some patients [7]. Organomegaly can present as hepatomegaly, splenomegaly, and lymphadenopathy. Endocrinopathy mainly presents as gonadal and adrenal axis abnormalities [8]. Skin changes include white nails, clubbing, hyperpigmentation, hemangiomas, hypertrichosis, acrocyanosis, sclerodermoid changes and rarely calciphylaxis [9]. Laboratory studies often show an absence of cytopenias with an estimated 50% showing erythrocytosis and thrombocytosis. Plasma and serum levels of VEGF are significantly elevated in POEMS syndrome and generally correlate with the disease activity of the syndrome. A recent review has demonstrated that a plasma VEGF level of above 200 pg/mL showed a specificity of 95% and sensitivity of 68% in those with POEMS syndrome [7]. Diagnosis of POEMS syndrome should be made by the coexistence of two mandatory criteria (polyneuropathy and a clonal plasma cell dyscrasia), at least one major criteria and at least one of the minor criteria (Figure 5) [7]. Endocrinopathy as one the minor diagnostic criteria does not include diabetes mellitus and thyroid abnormalities due to the high prevalence of these conditions and the criteria were designed to retain both sensitivity and specificity [8]. 

There are no published randomized trials among patients with POEMS syndrome and therefore treatment is based mainly on case reports and case series. Treatment is directed at the underlying monoclonal plasma cell disorder rather than anti-VEEF antibodies and whether patients have localized monoclonal disease versus those with disseminated bone marrow involvement. Localized plasma cell disease in the form of plasmacytomas without disseminated bone marrow involvement is treated with radiation [7]. With disseminated bone marrow involvement or those who have progression of their localized disease after three-six months of radiation, systemic chemotherapy is recommended [7]. Anti-VEGF antibody use is conflicting [6,10]. All symptoms and signs of the syndrome improve when the underlying plasma cell dyscrasia responds to therapy [7].

## Conclusions

POEMS syndrome should be considered when a unique constellation of symptoms is observed, especially when they accompany other nonspecific symptoms such as polyneuropathy, endocrinopathies, and organomegaly. Early identification of the features of the POEMS syndrome is critical for patients so that it can identify an underlying plasma cell dyscrasias and reduce the morbidity and mortality associated with the disease. As seen in our case, plasma cell leukemia which remains the most aggressive plasma cell proliferative disorder was diagnosed with POEMS syndrome being the only identifying feature.
